# Factors affecting out-of-pocket expenditures for chronic and acute illnesses in Bangladesh

**DOI:** 10.1371/journal.pone.0320429

**Published:** 2025-04-09

**Authors:** Jinat Jahan Khan, Farzana Sehrin, Zahidul Quayyum, Abdur Razzaque Sarker, Mohammad Shafiqur Rahman

**Affiliations:** 1 Centre of Excellence for Urban Equity and Health (CUEH), BRAC James P Grant School of Public Health, BRAC University, Dhaka, Bangladesh; 2 Bangladesh Institute of Development Studies (BIDS), Dhaka, Bangladesh; 3 Institute of Statistical Research and Training (ISRT), University of Dhaka, Dhaka, Bangladesh; Utkal University, INDIA

## Abstract

**Background:**

In the absence of universal healthcare protection, out-of-pocket (OOP) expenditures are the main source of healthcare financing in Bangladesh. This study assesses the disparities in the overall and the components of OOP expenditures among households with both chronic and acute illnesses compared to those having acute illnesses only. It also identifies factors influencing OOP expenditures over time and examines patterns related to various illness conditions.

**Materials and methods:**

Data from the Household Income and Expenditure Surveys (HIES) of 2016–17 and 2022 were used. A Log-linear Multiple Regression Model was employed to identify factors influencing OOP expenditures in households with different disease profiles.

**Results:**

The average OOP expenditures increased significantly from 2016 to 2022, even after inflation adjustments. Higher expenditures were observed in urban households BDT 939.34 in 2016 and BDT 1605.36 in 2022, and in households having both chronic and acute illness conditions with OOP expenditure of BDT 2290.43 and BDT 3525.32 in 2016 and 2022 respectively. Wealthier households spent more on healthcare, with the cost of medicines being the largest component with over 50% of total OOP expenditures. The regression analysis suggests that area of residence (urban vs rural), household size, level of education of the household head, the presence of elderly members (≥60 years), the number of employed members and sick household members, and hospitalisation of household members were mainly responsible for higher OOP expenditure.

**Conclusion:**

Our study provides valuable insights on the determinants of OOP expenditures over time, with a notable increase among households managing both chronic and acute illnesses, and in urban areas. Key contributors to increased expenditures include medicines, medical tests and surgery costs, with cancer causing higher expenses. This study recommends improving treatment protocols help reduce unnecessary prescriptions of medicine and investigations, and alleviate financial burdens of the vulnerable population.

## Introduction

Illnesses and diseases impose a significant financial burden on individuals and households, particularly in low- and middle-income countries where out-of-pocket (OOP) expenditures are substantial due to the absence of comprehensive health insurance systems. In 2020, approximately 12.9% of the global population (around one billion people) were expected to allocate at least 10% of their household budget to healthcare services [1]. About 87% of the world population, mostly in middle-income countries, experiences catastrophic OOP expenditures [1]. These expenditures, which are paid directly by individuals without reimbursement from public or private insurance, create financial barriers for lower-income households and exacerbate inequalities in healthcare access [2–4]. The high cost of healthcare often prevents people, especially from lower-income households, from accessing necessary services such as medical, paramedical, and hospital services, etc [5].

OOP healthcare expenditures encompass all costs incurred while receiving healthcare, including preventive, curative, long-term, and rehabilitative care from any provider for all household members. These costs cover consultation fees, medicines, vaccines, pharmaceutical preparations, and other health products. If these expenses are financed by household income, savings, loans, or remittances without third-party reimbursement, they are classified as OOP expenditures under the International Classification for Health Accounts (ICHA) [6]. In low-income countries, OOP expenditures are the predominant source of healthcare financing, frequently exceeding government spending and reflecting a regressive and unsustainable financing method reliant on households’ financial capacity [7,8]. Higher OOP expenditures increase the financial burden on households, worsening economic disparities [9,10].

Households in low- and middle-income countries face a disproportionately higher financial burden from OOP expenditures compared to other regions [3,4,11]. Over 11.7% of the global population, or approximately 808 million people, face catastrophic health expenditures, with over 1.4% (97 million) pushed into poverty due to these costs [5,12]. A study revealed increased catastrophic health expenditures among economically disadvantaged groups in Bangladesh, Bolivia, Guatemala, India, and the Seychelles, with Bangladesh, Haiti, and India showing a higher impact on poorer households [12]. Catastrophic health expenditures refer to healthcare costs that exceed a certain share of household expenditures, typically over 40% of non-food spending or 10% or 25% of total household consumption expenditures [5,13]. In South Asia, Bangladesh has the second-highest percentage of OOP expenditures in health after Afghanistan, with OOP expenditures constituting 74% of total health expenditures in Bangladesh, compared to 77.21% in Afghanistan [1]. Moreover, rural households in Bangladesh suffer more due to high out-of-pocket expenditures as they are more likely to sell their assets to afford healthcare services [14].

Excessive OOP expenditures while obtaining healthcare can deter individuals from seeking essential medical treatment, thereby reducing overall household welfare [15]. Poorer households often incur higher OOP expenditures as they tend to delay or forgo healthcare services [16]. Households with members suffering from chronic illnesses typically incur higher OOP expenditures due to the need for long-term medication and regular medical consultations. Conversely, acute medical conditions may require immediate and intensive care, resulting in high one-time costs that can be financially devastating for households, particularly poorer ones [17–20]. Studies conducted in Bangladesh showed that OOP expenditures for chronic health conditions, such as diabetes and cardiovascular diseases, are persistent and accumulate over time, contributing to catastrophic health expenditures [21,22]. Another study found that chronic diseases contribute to catastrophic health expenditures due to the prolonged and consistent medical care they require [22]. Studies also suggest that acute health conditions can incur high treatment costs, especially in households with children, where acute illnesses necessitate frequent consultations and medications in Bangladesh [23,24].

Previous studies have examined OOP expenditures and catastrophic health expenditures in Bangladesh [14,25–27], as well as the healthcare costs of chronic and acute health conditions separately [22–24]. Our study aims to explore OOP expenditures for households with both chronic and acute illnesses and those with acute conditions only, examining how these expenditures vary over time and across different diseases and illnesses. There is a rationale behind choosing these two types of households. We have focused on two types of households such as households with both chronic and acute illnesses and households with acute illnesses only, because many patients suffering from chronic diseases may experience episodes of acute illnesses as well. Thus, it would not be appropriate if we strictly focus on households with only chronic diseases. Moreover, acute illnesses such as common childhood diseases, diarrhoea, typhoid and so on sometimes require hospitalisation or expensive medication that may incur higher health expenditures. Therefore, it is important to observe households with only acute illnesses too. This study seeks to understand the disparities in OOP expenditures of households with different disease profiles, as varying healthcare infrastructures, and socioeconomic characteristics lead to different expenditure patterns. Additionally, we aim to identify the factors that significantly influence health expenditures to determine context-specific drivers of OOP expenditures across different households. This analysis will enhance our understanding of the prevalence of OOP expenditures in different settings and examine whether higher OOP expenditures necessarily correspond to negative outcomes.

This research is guided by two principal objectives. Firstly, it has assessed the urban-rural disparities in the overall and the components of OOP expenditures between households with both chronic and acute illness conditions and those with acute illnesses only over time in Bangladesh. It has also calculated disease-specific OOP expenditures on an individual level. Secondly, it has identified and analysed the factors influencing the OOP expenditures of households with different illness conditions.

## Materials and methods

### Data source

This study used data from the Household Income and Expenditure Surveys (HIES) conducted by Bangladesh Bureau of Statistics in 2016–17 and 2022 [28,29]. These surveys are nationally representative datasets that include extensive modules on household characteristics and expenditures. As these surveys are conducted in every five years and there are many changes in the questionnaire over time, we have selected the HIES 2016–17 and 2022 that represent the two most recent datasets available and are comparable in terms of our concerned variables. Details of HIES 2016–17 dataset and how to access the data can be found elsewhere [30]. However, HIES 2022 is purchased from the Bangladesh Bureau of Statistics, and the authors are authorised to use them for research purposes. For data access, the concerned authority can be contacted through the BBS website [31]. Both surveys used a two-stage stratified sampling design. The 2016 HIES surveyed 46,080 households across 2,304 PSUs from 20 strata. These strata include eight rural divisions, eight urban divisions, and four city corporations. In the first stage, 20 main strata of administrative areas such as divisions, urban-rural, and city corporations were considered. Primary sampling units (PSUs), referred to as enumeration areas, were randomly selected. In the second stage, households were systematically selected from each PSU [28]. On the other hand, the 2022 HIES included 14,400 households from 720 PSUs, with the framework comprising 24 strata, including eight rural divisions, eight urban divisions, and eight city corporations. The strata were selected in the first stage, and then PSUs were determined in the later stage [29].

### Outcome variable

Households’ OOP expenditures are the main outcome variable in this study. OOP expenditures are a composite metric that accounts for all direct medical and non-medical healthcare expenses, including prescription drugs, doctor visits, hospital stays, tests, clinic fees, and travel expenses for medical purposes [32]. In this study, OOP expenditures were extracted from the health module (Section 3) of the HIES datasets. For both inpatient and outpatient services, separate calculations are made for this metric. In order to ensure uniformity, inpatient costs—which have a 12-month recall term—are changed to a 30-day period to match outpatient care costs. The monthly OOP that is produced as a result offers a thorough understanding of the financial strain that healthcare expenses place on households.

To facilitate both descriptive and empirical analyses, the datasets were adjusted using relevant survey weights. This adjustment was necessary to compare the data across these two periods, which featured different sample sizes, and to discern changes in OOP expenditures. To compare these expenditures between 2016 and 2022, the 2016 expenditures were adjusted for inflation in 2022 using the Consumer Price Index (CPI). The formula used here is as follows [33,34]:


$${\text{Adjusted OOP}}_{2022}=\left(\frac{CPI_{2022}}{CPI_{2016}}\right)*{\text{OOP}}_{2016}$$

Here, Adjusted OOP_2022_ = Adjusted Out-of-Pocket Expenditures in 2022

OOP_2016_ = Out-of-Pocket Expenditures in 2016

CPI_2016_ = Consumer Price Index for medical care and health expenses in 2016

CPI_2022_ = Consumer Price Index for medical care and health expenses in 2022

Note that this study has used national CPI values for medical care and health expenses, not the general CPI values. The rationale behind choosing these specific medical sector-based values is that most of the costs incurred are related to medical expenses, e.g., medicines, consultation fees, etc. Here, the CPI values for medical care and health expenses are 206.70 in 2016–17 and 253.62 in 2021–22 [35,36]. The adjustment has allowed for a consistent comparison across different years and economic conditions.

In addition, for descriptive analysis, component-based outpatient OOP expenditures and inpatient OOP expenditures were calculated. For consistency, the component-based expenditures of inpatient services were converted to a monthly basis as the study aimed to measure the proportion of each component’s expenditure in the total monthly inpatient OOP expenditures. Moreover, disease-specific OOP expenditures on an individual level were also calculated, providing detailed insights into the financial burden that is associated with each illness conditions.

### Major explanatory variables

Primary explanatory factors include a variety of household characteristics, socioeconomic and demographic traits that could have an impact on OOP expenditures based on earlier studies [14,22–27]. The geographic location (rural or urban), the size of the household (number of people), the gender, and the degree of education (none, primary, secondary, and higher) of the household head are some of these variables. The study also takes into account the number of adults 60 years or older and children aged one year old or below in the home, in addition to the number of household members who work. In order to account for the possibility that a person may have both acute and chronic illnesses, the total number of household members with any illness is also given. Wealth quintiles, which range from the poorest to the richest families, are used to illustrate economic standing based on primary component analysis (PCA). Another important factor is hospitalisation of any household member, which indicates if any members of the household were admitted to the hospital during the last 12 months.

### Model specification

This study employed a log-linear multiple regression model to address the right-skewed nature of OOP expenditure data. The log-linear model transforms the dependent variable, OOP expenditures, using its natural logarithm, thereby normalizing the data and enhancing the robustness of the outcome measures. For additional robustness, a Poisson regression model was also considered, given its appropriateness for skewed data distributions [37–39]. Both models were initially applied, and the Akaike Information Criterion (AIC) and Bayesian Information Criterion (BIC) were used to evaluate model fit. Although AIC and BIC do not provide absolute thresholds for model adequacy, lower values are generally indicative of a better fit [40,41]. In our analysis, the log-linear model consistently yielded lower AIC and BIC values compared to the Poisson model. Consequently, the log-linear model was selected, utilizing the logarithmic transformation of the dependent variable for regression analysis. The model is specified as follows:


$$lny_{i}=\alpha +\sum_{i=1}^{n}\beta_{i}x_{i}+\varepsilon_{i}$$

where *lny*_*i*_ represents the natural logarithm of the OOP expenditures for household *i*, and *x*_*i*_ denotes the set of explanatory variables. For the log-linear regression analysis, only the nonzero OOP expenditures as well as households which have at least one illness-affected member (e.g., have either chronic or acute or both illness conditions) were considered. Three household categories included households with all kinds of illness-affected members, households with chronic and acute illnesses and households with acute illnesses only. Additionally, Analysis of Variance (ANOVA) tests were conducted to explore the relationship between the dependent and independent variables. A two-way ANOVA was employed to assess control variables across two time periods. The log-transformed OOP variable was assumed to follow a normal distribution, consistent with the assumptions of ANOVA.

## Results

### Descriptive statistics

Table 1 shows the summary statistics of household characteristics. Most surveyed households have only acute illness-affected members in both years. It was 75.62% in 2016 and 76.89% in 2022. The percentage of households with both chronic and acute illnesses was 24.38% and 23.11% in respective years. In 2016, a larger proportion of surveyed households were in rural areas, and most of these households had two to five family members and one employed member on average. In 2022, the proportion of rural and urban households were almost same. The average number of employed household members was two to five in this year. Surveyed households mostly had one sick member in 2016, which became two to five members in 2022. The sickness can be due to chronic or acute illnesses. Moreover, most household heads were male, having a secondary education level. There were 12.66% of households with at least one child aged one or below, and 27.16% of households with at least one adult aged 60 or above in 2016. These percentage amounts were 14.92% and 34.51% respectively in 2022. One noticeable thing is that households in the middle quintile have increased over time but households in the poorest and richest quintiles have slightly decreased. Furthermore, around 8% and 12% of surveyed households had at least one household member hospitalised in 2016 and 2022 respectively.

**Table 1:** Summary statistics of relevant household characteristics.

Year	2016	2022
Main household characteristics	Mean (%) and CI (95%)	Mean (%) and CI (95%)
**Households with different diseases**		
Households with chronic and acute illnesses	24.38 [22.18 - 26.56]	23.11 [21.42 - 24.81]
Households with acute illnesses only	75.62 [73.44 - 77.82]	76.89 [75.21 - 78.56]
**Area**		
Rural	69.65 [61.41 - 77.89]	50.57 [31.44 - 69.67]
Urban	30.35 [22.11 - 38.59]	49.43 [30.33 - 68.56]
**Household size**		
Only one household member	2.95 [2.66 - 3.23]	2.14 [1.81 - 2.48]
2-5	82.61 [81.13 - 84.10]	78.48 [75.97 - 80.99]
6-10	14.18 [12.66 - 15.71]	18.75 [16.26 - 21.24]
>10	0.26 [0.17 - 0.35]	0.63 [0.37 - 0.88]
**Gender of household head**		
Male	87.10 [85.96 - 88.24]	88.50 [86.80 - 90.21]
Female	12.90 [11.76 - 14.04]	11.50 [9.79 - 13.20]
**Education of household head**		
No education	0.99 [0.86 - 1.13]	0.29 [0.17 - 0.42]
Primary education	24.90 [23.41 - 26.39]	13.93 [12.21 - 15.65]
Secondary education	39.45 [38.28 - 40.62]	32.51 [30.03 - 35.00]
SSC/equivalent	14.03 [13.38 - 14.68]	16.79 [15.86 - 17.71]
HSC/equivalent	10.71 [10.00 - 11.42]	15.70 [14.29 - 17.11]
Graduate/Post graduate/Other tertiary education levels	9.92 [8.88 - 10.97]	20.79 [17.31 - 24.28]
**Number of children aged one or below**		
None	87.34 [86.61–88.06]	85.07 [83.93 - 86.21]
One or more	12.66 [11.94 - 13.38]	14.93 [13.79 - 16.06]
**Number of adults aged 60 or above**		
None	72.84 [71.63 - 74.06]	65.49 [63.73 - 67.25]
One or more	27.16 [25.94 - 28.37]	34.51 [32.75 - 36.27]
**Number of employed household members**		
Only one household member	75.12 [72.95 - 77.28]	67.02 [63.52 - 70.52]
2-5	24.84 [22.69 - 26.99]	32.90 [29.41 - 36.40]
>5	0.04 [0.01 - 0.07]	0.08 [0.03 - 0.13]
**Total number of sick household members**		
Only one household member	55.15 [51.92 - 58.38]	37.27 [34.60 - 39.94]
2-5	42.57 [39.65 - 45.48]	57.80 [55.46 - 60.14]
6-10	2.19 [1.56 - 2.83]	4.78 [4.01 - 5.55]
>10	0.09 [0.02 - 0.15]	0.15 [0.08 - 0.23]
**Wealth quintiles**		
Poorest	20.00 [16.23 - 23.78]	19.64 [15.16 - 24.12]
Poorer	21.44 [19.50 - 23.37]	20.64 [17.99 - 23.29]
Middle	18.66 [17.21 - 20.11]	21.50 [19.15 - 23.85]
Richer	20.31 [18.16 - 22.46]	19.02 [16.44 - 21.60]
Richest	19.59 [16.73 - 22.44]	19.20 [15.43 - 22.97]
**Hospitatisation of any household member**		
No	92.08 [91.71 - 92.44]	88.29 [87.63 - 88.94]
Yes	7.92 [7.55 - 8.29]	11.71 [11.06 - 12.37]

Here, the mean and Confidence Interval (CI) are given in percentage amounts.

The overall average monthly OOP expenditures are BDT 909.00 and BDT 1564.68 in 2016 and 2022. The OOPs for households with both chronic and acute illnesses in 2016 and 2022 were BDT 2290.43 and BDT 3525.32 respectively, at current prices. After inflation adjustments, the adjusted OOP expenditure in 2022 was found to be BDT 2810.35 on average. The mean health expenditures for households with acute illnesses only in 2016 and 2022 were BDT 1384.99 and BDT 2204.64 respectively. Moreover, the inflation-adjusted mean OOP expenditure for such households was BDT 1699.38. These findings indicate that the average OOP expenditures increased over time, even after adjusting for inflation (Table 2).

**Table 2:** Statistical distributions of monthly OOP expenditures of households across different types of areas and households.

Monthly Out-of-Pocket (OOP) Expenditures of households				
Factors	Categories	Year	N	Mean (BDT)
Overall	Overall	2016	46075	909.00
		2022	14395	1564.68
		Adjusted in 2022*		1115.34
Households with different disease profiles	Households with chronic and acute illnesses	2016	5889	2290.43
		2022	1769	3525.32
		Adjusted in 2022*		2810.35
	Households with acute illnesses only	2016	18266	1384.99
		2022	5885	2204.64
		Adjusted in 2022*		1699.38
Area	Urban	2016	13884	939.34
		2022	6899	1605.36
		Adjusted in 2022*		1152.56
	Rural	2016	31866	877.13
		2022	7057	1448.49
		Adjusted in 2022*		1076.23
Wealth quintiles	Poorest	2016	9038	660.43
		2022	2729	1267.16
		Adjusted in 2022*		810.35
	Poorer	2016	9690	773.41
		2022	2868	1294.33
		Adjusted in 2022*		948.97
	Middle	2016	8431	909.60
		2022	2987	1614.30
		Adjusted in 2022*		1116.08
	Richer	2016	9178	888.14
		2022	2643	1567.44
		Adjusted in 2022*		1089.75
	Richest	2016	8852	1278.83
		2022	2668	1922.30
		Adjusted in 2022*		1569.12

Note: To compare the OOP of 2022 with those of 2016, we have adjusted inflation for the OOP expenditures of 2016 into 2022.

*Adjusted in 2022 = 2016’s OOP expenditures adjusted in 2022 = (2022’s CPI/2016’s CPI)*2016’s OOP expenditures.

All values are given in Bangladeshi Taka (BDT).

Regardless of the year and adjustments, households with both chronic and acute conditions incurred higher OOP expenditures, likely due to the regular and long-term medical expenses required by individuals with chronic diseases. In both periods, monthly OOP expenditures were higher in urban areas compared to rural areas. Even after inflation adjustments, these expenditures exceeded the average rural OOP expenditures. Thus, higher OOP payments for urban households over time was observed. Table 2 also presents OOP expenditures categorised by wealth quintiles for 2016 and 2022. The mean health expenditures have increased in the latter period across all quintiles. The richest quintile consistently shows the highest OOP expenditures, indicating that wealthier households spend more on healthcare services in absolute terms, whereas households in the middle quintile is more likely spend on healthcare compared to richer households.

To better understand which components of OOP expenditures contributing the most to the overall expenditure, the distribution of OOP expenditures was analysed. For households with both chronic and acute conditions, the cost of medicines accounted for 56.07% of outpatient OOP expenditures in 2016 (n = 4,817) and 48.67% in 2022 (n = 1,636). The cost of tests or investigations was 24.17% in 2016 (n = 4,817) and increased to 32.87% in 2022 (n = 1,636). Analysis of the components of outpatient services for households with acute diseases only also revealed that a significant portion of the outpatient OOP expenditures were due to medicines. Specifically, the proportions of the cost of medicines in outpatient OOP expenditures was 63.89% and 58.61% for such households in respective years. In contrast, the percentage of the cost of tests or investigations for households with acute illnesses only was lower compared to households with both types of illnesses (Table 3).

**Table 3:** Distribution of OOP expenditures (at current prices) by components across different households.

Components	All illness-affected households				Households with chronic and acute illnesses				Households with acute illnesses only			
	2016 (N = 20364)		2022 (N = 7243)		2016 (N = 4817)		2022 (N = 1636)		2016 (N = 15541)		2022 (N = 5604)	
	Average	Percentage	Average	Percentage	Average	Percentage	Average	Percentage	Average	Percentage	Average	Percentage
**Outpatient services (During the last 30 days)**		**% of Outpatient OOP**		**% of Outpatient OOP**		**% of Outpatient OOP**		**% of Outpatient OOP**		**% of Outpatient OOP**		**% of Outpatient OOP**
Consultation fees (visit)	180.43	10.51%	238.47	10.59%	235.64	9.46%	317.85	10.09%	150.35	10.32%	203.82	10.58%
Cost of medicines	1041.12	60.65%	1240.88	55.08%	1396.3	56.07%	1533.49	48.67%	930.65	63.89%	1129.36	58.61%
Cost of tests/ investigation	333.60	19.43%	605.56	26.88%	601.94	24.17%	1035.59	32.87%	243.86	16.74%	458.57	23.80%
Transport cost	161.51	9.41%	167.82	7.45%	256.61	10.30%	264.07	8.38%	131.81	9.05%	135.17	7.01%
Total	1716.65	100.00%	2252.73	100.00%	2490.48	100.00%	3151.01	100.00%	1456.67	100.00%	1926.92	100.00%
	**2016 (N = 3120)**		**2022 (N = 1713)**		**2016 (N = 720)**		**2022 (N = 299)**		**2016 (N = 1556)**		**2022 (N = 873)**	
**Inpatient services (Calculated on a monthly basis)**		**% of Inpatient OOP**		**% of Inpatient OOP**		**% of Inpatient OOP**		**% of Inpatient OOP**		**% of Inpatient OOP**		**% of Inpatient OOP**
Surgery cost	4925.25	19.38%	8774.70	23.37%	4676.71	17.43%	10637.29	21.86%	4368.15	20.44%	6833.34	23.57%
Consultation fees (visit)	1316.70	5.18%	1922.67	5.12%	1486.28	5.54%	2696.25	5.54%	1129.06	5.28%	1568.00	5.41%
Bed/cabin charges	2255.85	8.88%	5266.46	14.03%	2267.21	8.45%	6858.36	14.09%	1855.92	8.69%	3658.51	12.62%
Cost of medicines	7592.48	29.87%	10149.82	27.03%	8786.70	32.75%	15291.49	31.42%	5968.71	27.93%	7910.34	27.29%
Cost of medical tests/investigations	3583.39	14.10%	6213.26	16.55%	3400.28	12.67%	7821.10	16.07%	2882.61	13.49%	4868.97	16.80%
Transport cost	1589.97	6.26%	2234.05	5.95%	1747.36	6.51%	2431.99	5.00%	1322.16	6.19%	1675.91	5.78%
Informal tips	857.04	3.37%	293.44	0.78%	1471.68	5.48%	310.00	0.64%	710.94	3.33%	201.12	0.69%
Other formal changes	1589.97	6.26%	1671.10	4.45%	1747.36	6.51%	1826.36	3.75%	1322.16	6.19%	1339.36	4.62%
Maternity costs	428.05	1.68%	770.58	2.05%	176.85	0.66%	736.79	1.51%	403.58	1.89%	653.95	2.26%
Midwife	275.05	1.08%	111.45	0.30%	267.84	1.00%	36.62	0.08%	270.58	1.27%	110.49	0.38%
Others	1001.93	3.94%	137.58	0.37%	804.07	3.00%	15.22	0.03%	1132.95	5.30%	166.59	0.57%
Total	25415.67	100.00%	37545.10	100.00%	26832.34	100.00%	48661.47	100.00%	21366.81	100.00%	28986.57	100.00%

Here, Outpatient OOP= Outpatient Out-of-Pocket expenditures and Inpatient OOP= Inpatient Out-of-Pocket expenditures

Average expenditures are given in Bangladeshi Taka (BDT).

When considering all households, regardless of the presence of different diseases, medicines contributed the highest to the outpatient OOP expenditures, followed by the cost of tests or investigations. The proportion of the cost of medicines in total outpatient expenditures was considerably higher in rural areas than urban areas. However, the percentages of consultation fees and the cost of tests and investigations in total outpatient OOP expenditures were higher for households in urban areas (S1 and S2 Table in S1 File). In rural areas, transport costs contributed to around 8–11% of the outpatient expenditures across different illness-affected households and time periods, whereas they varied from 6% to 10% in urban areas. In these areas, the distance from home to medical facilities as well as area-based cost of utilising public or private transportation or traffic jams potentially modified those proportions (S1 and S2 Table in S1 File).

Similar to outpatient services, the proportion of the medicine costs was the highest in the total inpatient OOP expenditures regardless of whether it was an urban or rural area. The second-largest contributor was the surgery costs in both urban and rural areas. The other three components of inpatient OOP expenditures that contribute the most are cost of medical tests/investigations, bed/cabin charges and consultation fees respectively. Moreover, it was also found that the contribution of cost of medicines in inpatient OOP expenditures is higher in rural areas than in urban areas but surgery costs and consultation fees contribute more to these expenses in urban areas (S1 and S2 Table in S1 File).

Now, among the chronic illness conditions, the highest total OOP expenditure was associated with cancer, averaging BDT 13690.29 per month in 2016 and over BDT 33668.06 in 2022 at current prices. Even after inflation adjustments, the expenditure of 2016 estimated at BDT 16797.93 in 2022 (Table 4). The second-highest OOP expenditures in 2016 resulted from kidney diseases, with expenditures of BDT 7749.77, which further increased to BDT 9508.93 after inflation adjustments in latter period. This average expenditure from kidney diseases was BDT 15782.93 in 2022. However, the second- and third-highest OOP expenditures incurred from the liver diseases and chronic heart diseases in 2022. The average expenditures were BDT 21183.02 and BDT 16186.79 respectively this year. Apart from these, paralysis is also one of the chronic conditions that incurred higher costs in both periods. In summary, the five chronic diseases such as cancer, kidney diseases, liver diseases, chronic heart diseases and paralysis mainly incurred the highest OOP expenditures in both periods. On the other hand, the lowest average OOP expenditure over time was detected for gastric/ulcer conditions. Arthritis and skin problems also incurred lower costs compared to other chronic diseases. For better understanding and comparison among these costs, S1 Figure in S1 File shows the detailed picture of the average monthly OOP expenditures by different types of chronic diseases.

**Table 4:** Average monthly OOP expenditures at current and adjusted prices by different types of chronic illness conditions.

Year	2016	2022	Adjusted in 2022
Chronic illness conditions	Average OOP expenditures	Average OOP expenditures	Adjusted average OOP expenditures in 2022
Chronic fever	1470.91	3060.38	1804.79
Injury/Disability	1742.57	2584.93	2138.12
Chronic Heart Disease	4924.81	16186.79	6042.72
Respiratory Diseases/Asthma/Bronchitis	2596.14	4955.33	3185.45
Gastric/ ulcer	1089.27	1498.16	1336.53
Hypertension	2934.90	4701.97	3601.10
Arthritis/ Rheumatism	1234.23	1795.27	1514.40
Skin problem	1104.51	1983.07	1355.22
Diabetes	1760.51	2404.08	2160.14
Cancer	13690.29	33668.06	16797.93
Kidney Diseases	7749.77	15782.93	9508.93
Liver Diseases	5819.44	21183.02	7140.43
Mental health	4433.42	7481.60	5439.79
Paralysis	5792.69	10429.58	7107.60
Ear/ ENT problems	3896.50	2860.86	4780.99
Eye problem	3218.11	6981.57	3948.61

Note: To compare the OOP of 2022 with those of 2016, we have adjusted inflation for the OOP expenditures of 2016 into 2022.

Here, Adjusted in 2022= Adjusted OOP expenditures in 2022 = (2022’s CPI/2016’s CPI)*2016’s OOP expenditures.

All values are given in Bangladeshi Taka (BDT).

We have also calculated average OOP expenditures for acute diseases, as presented in Table 5 and S2 Fig in S1 File. Note that some of these conditions may arise due to chronic illnesses; however, our focus is on acute conditions that occurred within the last 30 days and are not directly related to those chronic conditions already mentioned in Table 4. Among these acute conditions, pregnancy-related healthcare costs were the highest in both years. In 2016, pregnancy-related conditions had an average OOP expenditure of BDT 3722.98, and BDT 4568.08 after adjustments at current prices. By 2022, this monthly mean expense increased to around BDT 7296.47. It indicated a substantial real increase over time.

**Table 5:** Average monthly OOP expenditures at current and adjusted prices by different types of acute illness conditions.

Year	2016	2022	Adjusted in 2022
**Acute illness conditions**	**Average OOP expenditures**	**Average OOP expenditures**	**Adjusted average OOP expenditures in 2022**
Diarrhoea	1308.94	2305.12	1606.06
Fever	1310.05	2087.20	1607.42
Dysentery	1272.11	2920.30	1560.87
Pain	1941.39	2981.09	2382.08
Injury/Accident	2950.49	4299.65	3620.23
Weakness	1613.46	2817.95	1979.71
Pneumonia	2447.02	3165.41	3002.49
Typhoid	3119.35	5769.12	3827.43
Malaria	2017.18	3660.00	2475.08
Jaundice	2107.76	5248.76	2586.22
Female diseases	2804.75	4090.27	3441.41
Pregnancy related	3722.98	7296.47	4568.08
Scabies/skin diseases	1818.02	3366.52	2230.71

Note: To compare the OOP of 2022 with those of 2016, we have adjusted inflation for the OOP expenditures of 2016 into 2022.

Here, Adjusted in 2022= Adjusted 2022’s OOP= (2022’s CPI/2016’s CPI)*2016’s OOP.

All values are given in Bangladeshi Taka (BDT).

However, these average expenses are exclusively related to those women who were pregnant at those times. If we observe the OOP expenditures regardless of their gender, the highest expenditures were incurred due to typhoid, with average costs of BDT 3119.35 in 2016 and BDT 3827.43 after inflation adjustments. The average expenditure was BDT 5769.12 in 2022 at current prices. As per the Table 5, sudden injuries and accidents led to significant high OOP expenditures in both periods. On the contrary, acute illness conditions like diarrhoea, fever and dysentery had the lowest expenditures over the years. These conditions also show moderate increases when these are adjusted for inflation.

### Statistical models

Table 6 presents the results of the log-linear regression analysis, examining the determinants of OOP expenditures for households which have at least one illness-affected member (e.g., have either chronic or acute or both illness conditions) and have made some health expenses. Before that, we have run ANOVA tests, and all results were compiled in S3 Table in S1 File. These results indicate all the selected independent variables except the gender of the household head are significantly related to the dependent variable, although some categories, such as the education of the household head, the number of employed household members, and wealth quintiles, showed some insignificant categories in any of the periods.

**Table 6:** Results from Log-linear models for out-of-pocket (OOP) expenditures.

Dependent variable= The log form of out-of-pocket expenditures	All illness-affected households		Households with chronic and acute illnesses		Households with acute illnesses only	
	2016	2022	2016	2022	2016	2022
Area (Base = Rural)						
Urban	0.081**	0.114***	0.042	0.126	0.104***	0.105**
	[0.014 – 0.149]	[0.029 – 0.198]	[-0.132 – 0.216]	[-0.093 – 0.346]	[0.032 – 0.175]	[0.012 – 0.198]
Household size	0.040***	0.021	0.132***	0.098**	0.022**	0.017
	[0.018 – 0.062]	[-0.008 – 0.050]	[0.089 – 0.176]	[0.023 – 0.173]	[0.001 – 0.044]	[-0.132 – 0.049]
Gender of household head (Base = Male)						
Female	0.145***	0.123*	-0.040	0.124	0.215***	0.092
	[0.064 – 0.227]	[-0.008 – 0.254]	[-0.226 – 0.146]	[-0.191 – 0.440]	[0.128 – 0.302]	[-0.051 – 0.235]
Education of household head	0.116***	0.078***	0.132***	0.102**	0.112***	0.061***
	[0.089 – 0.141]	[0.047 – 0.110]	[0.072 – 0.191]	[0.019 – 0.184]	[0.083 – 0.141]	[0.026 – 0.096]
Number of children aged one or below (Base = None)						
One or more	0.002	0.121***	0.077	0.214	0.023	0.112**
	[-0.064 – 0.069]	[0.031 – 0.211]	[-0.128 – 0.284]	[-0.136 – 0.565]	[-0.047 – 0.194]	[0.017 – 0.207]
Number of adults aged 60 or above (Base = None)						
One or more	0.111***	-0.029	0.078	0.179*	0.121***	-0.107**
	[0.043 - 0.179]	[-0.115 – 0.055]	[-0.052 – 0.209]	[-0.022 – 0.379]	[0.049 – 0.194]	[-0.202 – -0.012]
Number of employed household members	-0.079***	-0.132***	-0.139***	-0.152**	-0.053**	-0.143***
	[-0.119 – -0.039]	[-0.182 – -0.082]	[-0.224 – -0.054]	[-0.285 – -0.018]	([-0.095 – -0.009]	[-0.196 – -0.089]
Total number of sick household members	0.199***	0.267***	0.015	0.173***	0.218***	0.272***
	[0.182 – 0.217]	[0.245 – 0.289]	[-0.028 – 0.058]	[0.101 – 0.245]	[0.199 – 0.237]	[0.248 – 0.296]
Wealth quintiles (Base = Poorest)						
Poorer	0.021	0.037	0.051	0.257*	0.014	-0.014
	[-0.054– 0.095]	[-0.075 – 0.149]	[-0.126 – 0.228]	[-0.045 – 0.559]	[-0.066 – 0.095]	[-0.133 – 0.106]
Middle	0.118***	0.246***	0.131	0.318**	0.117***	0.249***
	[0.042 – 0.193]	[0.134 – 0.358]	[-0.053 – 0.315]	[0.032 – 0.603]	[0.036 – 0.197]	[0.125 – 0.374]
Richer	0.053	0.156**	0.016	0.429***	0.062	0.124*
	[-0.031 - 0.138]	[0.031 – 0.276]	[-0.186 – 0.218]	[0.123 – 0.735]	[-0.024 – 0.147]	[-0.007 – 0.255]
Richest	0.053	0.201***	0.174	0.552***	0.012	0.089
	[-0.039 – 0.144]	[0.071 – 0.331]	[-0.044 – 0.392]	[0.226 – 0.879]	[-0.087 – 0.111]	[-0.053 – 0.232]
Hospitatisation of any household member (Base = No)						
Yes	1.112***	1.388***	1.144***	1.411***	1.179***	1.464***
	[1.035 – 1.189]	[1.302 – 1.475]	[1.002 – 1.287]	[1.196 – 1.626]	[1.091 – 1.269]	[1.359 – 1.568]
Constant	5.450***	5.459***	5.897***	5.373***	5.395***	5.526***
	[5.344 – 5.556]	[5.307 – 5.612]	[5.645 – 6.149]	[4.967 – 5.779]	[5.286 – 5.505]	[5.362 – 5.692]
Observations	19752	7751	4214	1568	14753	5645
R-squared	0.158	0.249	0.118	0.194	0.178	0.278

95% confidence intervals are in parenthesis; *** p < 0.01, ** p < 0.05, * p < 0.1

In this study, we have found that the area of residence, household size, the education of household head, the number of adults aged 60 or above, the number of employed household members, total number of sick household members and hospitalisation of any household member are mostly significant factors that influence the OOP expenditures across different households and periods. As per the findings of this study, urban households with illness-affected members consistently spent on healthcare services in both periods. Even households with acute illnesses only incurred higher OOP expenditures if they resided in urban areas. Household size is associated with OOP expenditures positively and significantly only in 2016. However, this predictor shows a persistent positive significance for households with both chronic and acute illnesses, suggesting that larger households with multiple health issues are more likely to face a higher burden of medical services or treatment.

We observed that female-headed households spent significantly and positively more on health in both periods. But the gender of household is not a significant factor for two specific household categories. Education of household head and the increasing number of sick household members depicted as significant determinants of OOP expenditures across all household categories and years. Age demographics also play an important role in the case of OOP expenditures. For instance, the presence of young children aged one or below and adults aged 60 or above impact healthcare needs, and eventually healthcare costs. The presence of young children was a significant factor of increasing OOP expenditures in 2022 for all illness-affected households as well as households with acute illnesses only. For households affected by both chronic and acute illnesses, the presence of an older adult is associated with an increase in OOP expenditures. In contrast, the presence of such elderly members appears to increase health expenditures even for households with only acute illnesses in both periods. It is quite plausible that the number of employed household members is negatively associated with health expenditures across all household categories and years. In other words, employment reduces these health expenditures significantly over time. On the contrary, wealthier households consistently spent more on healthcare compared to the poorest households. The impact of wealth quintiles is even more substantial in 2022, especially for households with both chronic and acute illnesses. The disparity among different wealth quintiles is quite evident in this study. Furthermore, hospitalisation of at least one household member is a significant driver to increase OOP expenditures across all household categories and time periods. The effect of this determinant is more significant for households dealing with both chronic and acute illness conditions.

## Discussion

Bangladesh faces significant challenges in its health systems, while experiencing a double burden of diseases with low service coverage aggravated by lack of effective financial risk protection mechanism. The country’s healthcare system is still developing its essential conditions, needed to achieve the Universal Health Coverage (UHC) that needs to be attained by 2030 [15]. Bangladesh is signatory to the Sustainable Development Goals (SDGs), where the third goal is “Ensure healthy lives and promote well-being for all at all ages”, and the eighth target for this goal states, “Achieve UHC, including financial risk protection, access to quality essential healthcare services and access to safe, effective, quality and affordable essential medicines and vaccines for all” [42]. Today, UHC has not been fully implemented, as a result, OOP expenditures often fill the gap left by limited or non-existent public healthcare provisions. It is essential to understand the components of OOP expenditures to identify policy interventions that can protect vulnerable populations from excessive financial burden.

This study provides a comprehensive analysis of OOP expenditures across households with chronic and acute illness as well as their components over two time periods (2016 and 2022). Unlike previous studies [14,22–27] which focused on aggregate OOP expenditures or urban-rural disparities, this study has analysed the cost drivers such as medicines, diagnostics, inpatient stay and other indirect payments, and evaluate their impact on households with different illness conditions. By identifying these drivers, this study highlights areas where targeted interventions could reduce costs and help fulfil one of the main conditions for achieving UHC. For instance, ensuring the availability of affordable prescription medicines and expanding diagnostic facilities in public hospitals can reduce the financial burden on households. Furthermore, enhancing the quality of services in public hospitals can decrease reliance on private healthcare providers, and improve the accessibility and affordability of healthcare, especially for resource-poor households.

The findings reveal persistent rise in OOP expenditures across all household types over two periods, even after inflation adjustments. Households managing both chronic and acute illness incur higher financial burdens compared to those dealing with acute conditions alone. Chronic diseases such as cancer, kidney conditions were among the most expensive ones, reflecting prolonged and specialised care. For acute conditions, pregnancy-related healthcare and typhoid were the costliest, with expenditures for treating typhoid increasing significantly over time. Sudden injuries and accidents also accounted for substantial expenses, while diarrhoea, fever, and dysentery were associated with lowest costs. These findings align with earlier studies showing that households with chronic or non-communicable diseases are more likely to experience catastrophic expenditures pushing families to poverty [14,21,22]. Medicines remain the largest contribution to OOP expenditures across all settings, followed by diagnostics and surgical costs. This may be the result of unnecessary prescription and medicines and investigations [14,43]. Urban households incur higher expenditures on consultation fees and medical tests, likely due to better access to specialist services and diagnostic facilities. In contrast, rural households depend heavily on prescribed medicines, reflecting limited availability of diagnostic services. These findings align with prior studies emphasizing the disproportionate burden of health expenses on urban households [11,14,43].

Several household characteristics significantly influence OOP expenditures in this study. Urban residence, larger household size, and higher education level of household heads are associated with increased healthcare spending. Households with higher number of members may face higher expenses due to diverse healthcare needs of members from different age groups, e.g., young children requiring vaccinations and older adults requiring regular medical attention for chronic illnesses [43–45]. This study found that female-headed households also report higher OOP expenditures which consistent with findings that suggest higher healthcare utilisation and reporting of illness in these households [46,47]. However, some studies found non-significant association between household head gender and OOP expenditures [8,44], while others indicate that gender significantly influences household healthcare spending patterns [48,49]. Households with educated heads tend to incur higher healthcare expenditures, likely due to better health awareness, higher income, and the ability to afford more expensive care [18,50,51]. However, these expenses decrease with the number of employed household members. The number of wage earners typically decreases the likelihood of incurring OOP expenditures. Previously, it was also found that the higher number of wage earners is associated with less health expenditures as these earners are more likely to be healthier and have better access to preventive care [52,53]. Hospitalisation is a significant driver of OOP expenditures as households with at least one hospitalised member incur considerably higher costs due to treatment and extended stays. This aligns with earlier studies indicating that hospitalisation leads to excessive healthcare spending [8,54]. A well-functioning and efficient referral system would prioritise emergency medical cases, and prevent unnecessary hospitalisations and OOP expenditures [55,56]. In a referral system, a health worker at one specific level of the health system seeks the assistance of another worker at a differently resourced facility to manage a clinical condition [57]. Manageable or acute health conditions may be treated at the primary healthcare facilities in this approach so that the tertiary healthcare facilities or hospitals do not get burdened with all kinds of patients, and can properly focus on emergency and complex cases. Referral efficiencies will also reduce delays in medical treatments and diversion of patients by middlemen inappropriately, and increase service quality and will reduce OOP expenditures.

Wealthier households incur higher absolute healthcare costs reflecting their ability to access quality services, whereas poorer households face a disproportionately greater financial burden relative to their income [14,22]. Limited income, coupled with high OOP expenditures, often restrict poorer households taking modern healthcare. Patients in such households may sometimes opt for traditional healthcare or unsafe care, increasing their health risks [58]. Implementing social safety nets with a special focus on medical care may help address these inequities and enhance access to care for financially vulnerable groups. Ultimately, this could steer the country closer to achieving universal health coverage.

## Limitations

There are some limitations of this study. As we wanted to compare the OOP expenditures of 2016 and 2022, we have selected the health module of the HIES dataset that mainly represents health costs on a monthly basis though OOP expenditures are often calculated on a yearly basis. Moreover, one of our targets was to analyse these OOP expenditures by components. In that case, we again needed to rely on the health module that has recorded these costs on a 30-day recall mostly. Since the episodes of illness conditions happened during the last 30-day period, we have focused on monthly OOP expenditures for consistency and coherence rather than yearly OOP expenditures. Consequently, it may be plausible that the overall average health expenditures are slightly lower than those found in HIES reports as we have only considered disease episode-wise costs (during the last 30 days), and did not focus on yearly health expenditures from the consumption module that include some other expenditures like costs of therapy accessories or equipment on a yearly basis. Despite these limitations, this study shows the complex scenario of OOP expenditures for households with different disease profiles and geographical areas in Bangladesh, highlighting its varying magnitude and impact on different contexts. A useful aspect in our methods is the use of Consumer Price Index (CPI) values specific to medical care for inflation adjustment, ensuring a more accurate analysis of expenditures. This approach enhances the validity of the findings, and offers insights for future research on healthcare costs in Bangladesh.

## Conclusion

In light of the findings of this study, some policy recommendations emerge to address the negative outcomes or challenges of increasing OOP expenditures. One is to develop interventions that support households affected by chronic conditions, mostly with non-communicable disease, including an updated fair pricing system of essential medicines for all and extended insurance coverage for chronic diseases. Note that fair price is the price that balances the interest and welfare of both patients and manufacturers of medicines. Another way is improving and implementing existing treatment protocols that may help reducing unnecessary prescriptions of medicine and investigations. Affordable prescription medicines and expanded diagnostic facilities in public hospitals are also required to make healthcare services accessible and affordable for everyone. Furthermore, efficient referral systems and social safety nets may improve healthcare accessibility, enhance service quality and reduce healthcare inequities.

## Supporting information

S1 File Supporting Information. (DOCX)
