# Supplementary material for: Factors affecting out-of-pocket expenditures for chronic and acute illnesses in Bangladesh
Source: PLoS One. 2025 Apr 9;20(4):e0320429. doi: 10.1371/journal.pone.0320429 (PMC11981217; doi:10.1371/journal.pone.0320429)

**Supplementary Tables**

**Table S1.** Distribution of OOP expenditures (at current prices) by components across different households in urban areas

| **Components** | **All illness-affected households** | | | | **Households with chronic and acute illnesses** | | | | **Households with acute illnesses only** | | | |
| --- | --- | --- | --- | --- | --- | --- | --- | --- | --- | --- | --- | --- |
|  | **2016 (N = 5909)** | | **2022 (N = 3488)** | | **2016 (N = 1250)** | | **2022 (N = 761)** | | **2016 (N = 4658)** | | **2022 (N = 2727)** | |
|  | **Average** | **Percentage** | **Average** | **Percentage** | **Average** | **Percentage** | **Average** | **Percentage** | **Average** | **Percentage** | **Average** | **Percentage** |
| **Outpatient services (During the last 30 days)** |  | **% of Outpatient OOP** |  | **% of Outpatient OOP** |  | **% of Outpatient OOP** |  | **% of Outpatient OOP** |  | **% of Outpatient OOP** |  | **% of Outpatient OOP** |
|  |  |  |  |  |  |  |  |  |  |  |  |  |
| Consultation fees (visit) | 203.14 | 11.33% | 260.32 | 11.24% | 263.99 | 9.80% | 371.75 | 10.21% | 186.85 | 12.05% | 229.22 | 11.78% |
| Cost of medicines | 1044.37 | 58.26% | 1209.44 | 52.21% | 1421.52 | 52.78% | 1707.88 | 46.88% | 942.95 | 60.82% | 1070.35 | 54.99% |
| Cost of tests/investigation | 391.67 | 21.85% | 698.10 | 30.14% | 750.09 | 27.85% | 1314.51 | 36.09% | 295.31 | 19.05% | 526.08 | 27.03% |
| Transport cost | 153.28 | 8.55% | 148.55 | 6.41% | 257.50 | 9.56% | 248.56 | 6.82% | 125.27 | 8.08% | 120.65 | 6.20% |
| Total | 1792.46 | 100.00% | 2316.41 | 100.00% | 2693.10 | 100.00% | 3642.70 | 100.00% | 1550.39 | 100.00% | 1946.29 | 100.00% |
|  | **2016 (N = 1083)** | | **2022 (N = 914)** | | **2016 (N = 207)** | | **2022 (N = 162)** | | **2016 (N = 577)** | | **2022 (N = 453)** | |
| **Inpatient services (Calculated on a monthly basis)** |  | **% of Inpatient OOP** |  | **% of Inpatient OOP** |  | **% of Inpatient OOP** |  | **% of Inpatient OOP** |  | **% of Inpatient OOP** |  | **% of Inpatient OOP** |
| Surgery cost | 5631.49 | 20.37% | 9426.42 | 23.99% | 5801.19 | 17.50% | 11389.20 | 21.84% | 4603.35 | 20.88% | 8232.23 | 28.68% |
| Consultation fees (visit) | 1407.09 | 5.09% | 1994.53 | 5.08% | 2174.04 | 6.56% | 2386.08 | 4.58% | 1105.93 | 5.02% | 1686.88 | 5.88% |
| Bed/cabin charges | 2556.98 | 9.25% | 6141.40 | 15.63% | 3018.60 | 9.10% | 7724.26 | 14.81% | 2078.97 | 9.43% | 3840.81 | 13.38% |
| Cost of medicines | 8488.96 | 30.70% | 10143.56 | 25.81% | 11437.35 | 34.49% | 16166.27 | 31.00% | 6673.96 | 30.27% | 7095.05 | 24.72% |
| Cost of medical tests/investigations | 3942.66 | 14.26% | 6296.28 | 16.02% | 3516.25 | 10.60% | 8495.06 | 16.29% | 3094.29 | 14.03% | 4453.84 | 15.52% |
| Transport cost | 1582.02 | 5.72% | 2138.66 | 5.44% | 2021.58 | 6.10% | 2381.82 | 4.57% | 1262.46 | 5.73% | 1463.60 | 5.10% |
| Informal tips | 800.89 | 2.90% | 335.82 | 0.85% | 841.26 | 2.54% | 391.60 | 0.75% | 763.83 | 3.46% | 216.46 | 0.75% |
| Other formal changes | 1582.02 | 5.72% | 1591.24 | 4.05% | 2021.58 | 6.10% | 2112.41 | 4.05% | 1262.46 | 5.73% | 923.33 | 3.22% |
| Maternity costs | 540.77 | 1.96% | 991.47 | 2.52% | 133.33 | 0.40% | 1066.05 | 2.04% | 533.36 | 2.42% | 605.52 | 2.11% |
| Midwife | 320.28 | 1.16% | 114.39 | 0.29% | 847.10 | 2.55% | 14.20 | 0.03% | 122.89 | 0.56% | 81.68 | 0.28% |
| Others | 795.40 | 2.88% | 123.82 | 0.32% | 1344.93 | 4.06% | 24.69 | 0.05% | 548.03 | 2.49% | 102.36 | 0.36% |
| Total | 27648.54 | 100.00% | 39297.57 | 100.00% | 33157.21 | 100.00% | 52151.64 | 100.00% | 22049.54 | 100.00% | 28701.75 | 100.00% |
| ***Here, Outpatient OOP= Outpatient Out-of-Pocket expenditures and Inpatient OOP= Inpatient Out-of-Pocket expenditures*** | | | | | | | | | | | | |
| ***Average expenditures are given in Bangladeshi Taka (BDT).*** | | | | | | | | | | | | |

**Table S2.** Distribution of OOP expenditures (at current prices) by components across different households in rural areas

| **Components** | **All illness-affected households** | | | | **Households with chronic and acute illnesses** | | | | **Households with acute illnesses only** | | | |
| --- | --- | --- | --- | --- | --- | --- | --- | --- | --- | --- | --- | --- |
|  | **2016 (N = 14452)** | | **2022 (N = 3752)** | | **2016 (N = 3567)** | | **2022 (N = 875)** | | **2016 (N = 10883)** | | **2022 (N = 2877)** | |
|  | **Average** | **Percentage** | **Average** | **Percentage** | **Average** | **Percentage** | **Average** | **Percentage** | **Average** | **Percentage** | **Average** | **Percentage** |
| **Outpatient services (During the last 30 days)** |  | **% of Outpatient** |  | **% of Outpatient** |  | **% of Outpatient** |  | **% of Outpatient** |  | **% of Outpatient** |  | **% of Outpatient** |
|  |  | **OOP** |  | **OOP** |  | **OOP** |  | **OOP** |  | **OOP** |  | **OOP** |
| Consultation fees (visit) | 157.20 | 9.45% | 201.02 | 9.58% | 225.70 | 9.33% | 270.97 | 9.95% | 134.73 | 9.51% | 179.75 | 9.42% |
| Cost of medicines | 1039.37 | 62.46% | 1231.12 | 58.66% | 1387.46 | 57.35% | 1381.82 | 50.74% | 925.39 | 65.33% | 1185.29 | 62.10% |
| Cost of tests/investigation | 302.89 | 18.20% | 487.50 | 23.23% | 550.02 | 22.73% | 793.02 | 29.12% | 221.84 | 15.66% | 394.58 | 20.67% |
| Transport cost | 164.65 | 9.89% | 178.94 | 8.53% | 256.29 | 10.59% | 277.57 | 10.19% | 134.61 | 9.50% | 148.94 | 7.80% |
| Total | 1664.11 | 100.00% | 2098.58 | 100.00% | 2419.48 | 100.00% | 2723.37 | 100.00% | 1416.56 | 100.00% | 1908.56 | 100.00% |
|  | **2016 (N = 2036)** | | **2022 (N = 797)** | | **2016 (N = 513)** | | **2022 (N = 137)** | | **2016 (N = 979)** | | **2022 (N = 420)** | |
| **Inpatient services (Calculated on a monthly basis)** |  | **% of Inpatient OOP** |  | **% of Inpatient OOP** |  | **% of Inpatient OOP** |  | **% of Inpatient OOP** |  | **% of Inpatient OOP** |  | **% of Inpatient OOP** |
| Surgery cost | 4537.27 | 19.27% | 7822.21 | 22.25% | 4222.97 | 17.39% | 9748.18 | 21.89% | 4229.52 | 20.17% | 5324.53 | 18.18% |
| Consultation fees (visit) | 1239.79 | 5.27% | 1835.06 | 5.22% | 1208.76 | 4.98% | 3063.03 | 6.88% | 1142.69 | 5.45% | 1439.77 | 4.91% |
| Bed/cabin charges | 2096.78 | 8.91% | 4210.56 | 11.98% | 1964.02 | 8.09% | 5834.45 | 13.10% | 1724.46 | 8.23% | 3461.89 | 11.82% |
| Cost of medicines | 6800.09 | 28.89% | 10120.99 | 28.79% | 7717.14 | 31.78% | 14257.08 | 32.01% | 5553.06 | 26.49% | 8789.69 | 30.01% |
| Cost of medical tests/investigations | 3099.34 | 13.17% | 6078.44 | 17.29% | 3353.49 | 13.81% | 7024.16 | 15.77% | 2757.85 | 13.15% | 5316.72 | 18.15% |
| Transport cost | 1576.31 | 6.70% | 2321.44 | 6.60% | 1636.72 | 6.74% | 2491.31 | 5.59% | 1357.34 | 6.47% | 1904.89 | 6.50% |
| Informal tips | 884.15 | 3.76% | 244.07 | 0.69% | 1726.06 | 7.11% | 213.50 | 0.48% | 679.77 | 3.24% | 184.57 | 0.63% |
| Other formal changes | 1576.31 | 6.70% | 1758.11 | 5.00% | 1636.72 | 6.74% | 1488.10 | 3.34% | 1357.34 | 6.47% | 1788.07 | 6.10% |
| Maternity costs | 368.30 | 1.56% | 504.14 | 1.43% | 194.41 | 0.80% | 347.45 | 0.78% | 327.09 | 1.56% | 706.19 | 2.41% |
| Midwife | 251.14 | 1.07% | 104.59 | 0.30% | 34.10 | 0.14% | 63.14 | 0.14% | 357.62 | 1.71% | 141.57 | 0.48% |
| Others | 1112.28 | 4.72% | 151.20 | 0.43% | 585.83 | 2.41% | 4.01 | 0.01% | 1477.69 | 7.05% | 235.85 | 0.81% |
| Total | 23541.75 | 100.00% | 35150.79 | 100.00% | 24280.2 | 100.00% | 44534.42 | 100.00% | 20964.43 | 100.00% | 29293.76 | 100.00% |
| ***Here, Outpatient OOP= Outpatient Out-of-Pocket expenditures and Inpatient OOP= Inpatient Out-of-Pocket expenditures*** | | | | | | | | | | | | |
| ***Average expenditures are given in Bangladeshi Taka (BDT).*** | | | | | | | | | | | | |

**Table S3.** Results from Analysis of Variance (ANOVA) tests

| Dependent variable = **The log form of out-of-pocket (OOP) expenditures** | **Year** | |
| --- | --- | --- |
|  | **2016** | **2022** |
| **Area** (Base= Rural) |  |  |
| Urban | 0.08*** | 0.04*** |
|  | (0.02) | (0.04) |
| **Household size** (Base = Only one household member) |  |  |
| 2-5 | 0.56*** | 0.76*** |
|  | (0.07) | (0.15) |
| 6-10 | 0.87*** | 1.10*** |
|  | (0.07) | (0.15) |
| >10 | 1.24*** | 1.63*** |
|  | (0.18) | (0.24) |
| **Gender of household head** (Base = Male) |  |  |
| Female | -0.05 | -0.05 |
|  | (0.03) | (0.06) |
| **Education of household head** (Base = No education) |  |  |
| Primary education | 0.19* | 0.05 |
|  | (0.11) | (0.31) |
| Secondary education | 0.36*** | 0.21 |
|  | (0.11) | (0.31) |
| SSC/equivalent | 0.55*** | 0.36 |
|  | (0.11) | (0.31) |
| HSC/equivalent | 0.64*** | 0.43* |
|  | (0.11) | (0.31) |
| Graduate/Post graduate/Other tertiary education levels | 0.83*** | 0.63** |
|  | (0.11) | (0.31) |
| **Number of children aged one or below** (Base= None) |  |  |
| One or more | 0.19*** | 0.42*** |
|  | (0.03) | (0.04) |
| **Number of adults aged 60 or above** (Base= None) |  |  |
| One or more | 0.27*** | 0.22*** |
|  | (0.02) | (0.04) |
| **Number of employed household members** (Base=Only one household member) |  |  |
| 2-5 | 0.09*** | 0.05 |
|  | (0.02) | (0.04) |
| >5 | 1.07 | -0.23 |
|  | (0.51) | (0.51) |
| **Total number of sick household members** (Base = Only one household member) |  |  |
| 2-5 | 0.44*** | 0.51*** |
|  | (0.02) | (0.05) |
| 6-10 | 1.37*** | 1.46*** |
|  | (0.05) | (0.07) |
| >10 | 1.84*** | 2.12*** |
|  | (0.26) | (0.33) |
| **Wealth quintiles** (Base = Poorest) |  |  |
| Poorer | 0.05* | -0.07 |
|  | (0.03) | (0.05) |
| Middle | 0.17*** | 0.25*** |
|  | (0.03) | (0.05) |
| Richer | 0.16*** | 0.19*** |
|  | (0.03) | (0.06) |
| Richest | 0.38*** | 0.40*** |
|  | (0.03) | (0.06) |
| **Hospitatisation of any household member (Base = No)** |  |  |
| Yes | 1.21*** | 1.37*** |
|  | (6.35) | (6.53) |
| Observations | 45753 | 13959 |
| ***Standard errors in parenthesis; *** p < 0.01, ** p < 0.05, * p < 0.1*** | | |

**Supplementary Figures**

**Fig S1.** Average monthly OOP expenditures by different types of chronic illness conditions


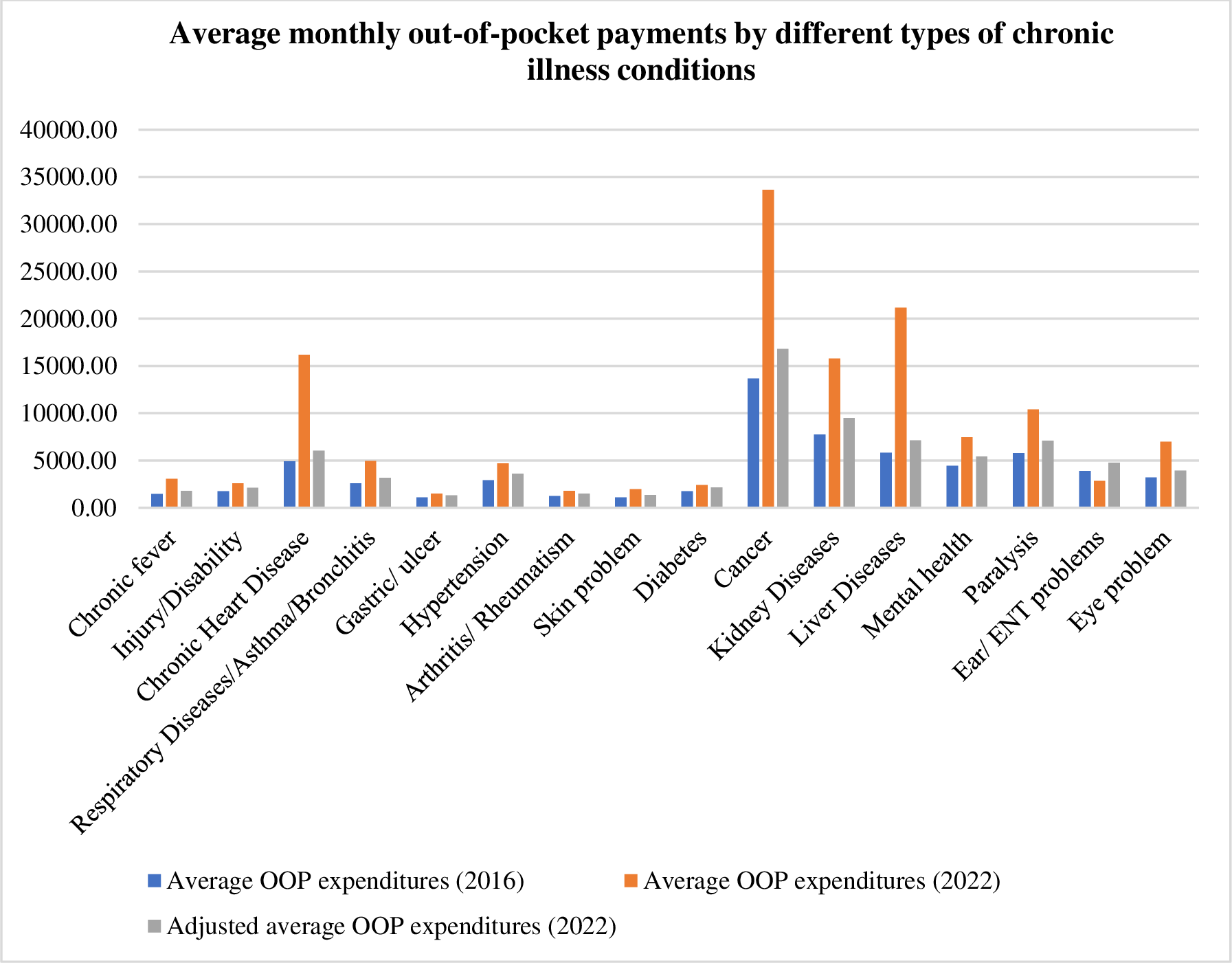


**Fig S2.** Average monthly OOP expenditures by different types of acute illness conditions


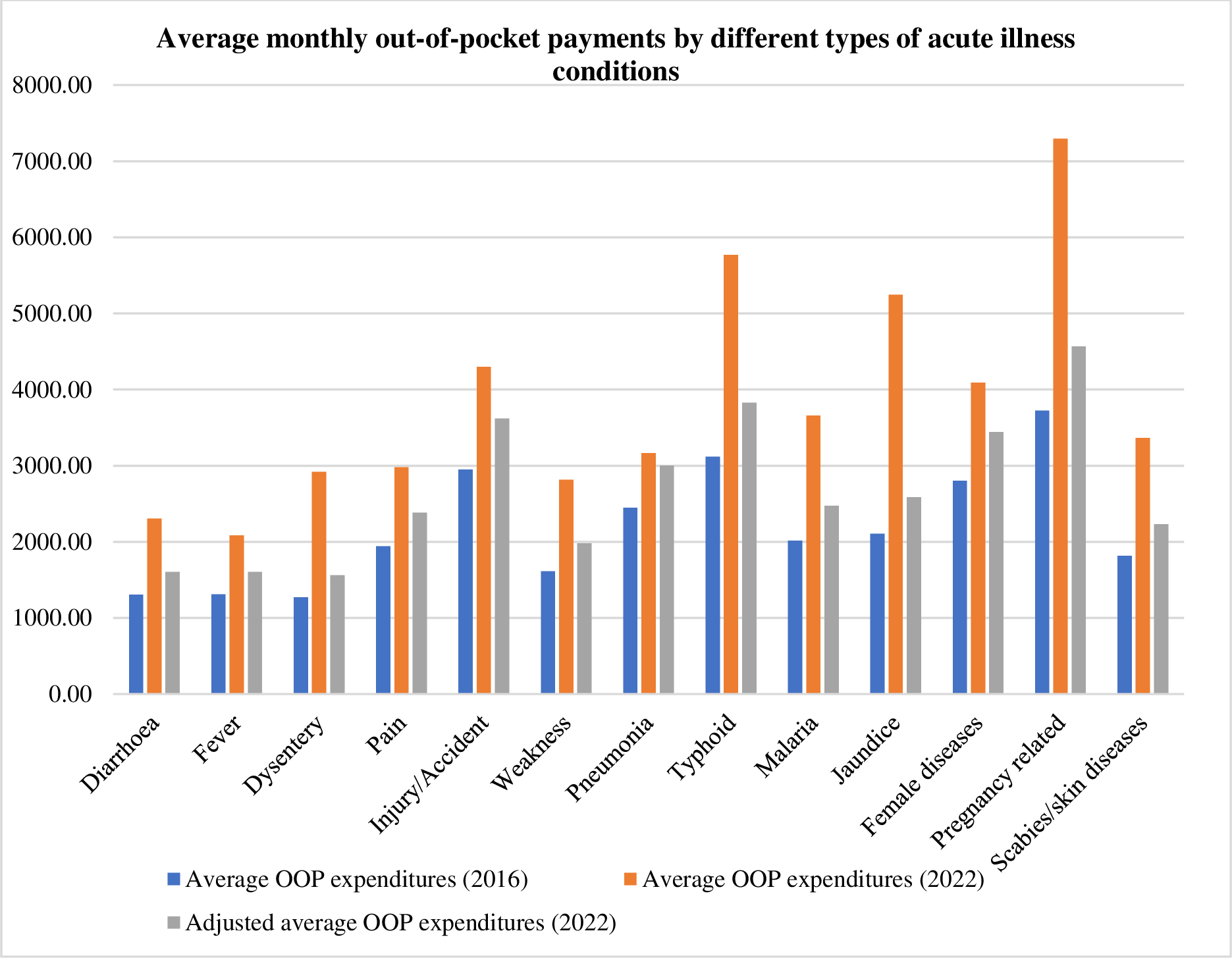

Supplement: S1 File — (DOCX) [file pone.0320429.s001.docx]
